# Adolescents’ physical activity during and beyond the Covid-19 pandemic: a qualitative study exploring the experiences of young people living in the context of socioeconomic deprivation

**DOI:** 10.1186/s12889-024-19777-z

**Published:** 2024-10-22

**Authors:** Olivia Alliott, Hannah Fairbrother, Esther van Sluijs

**Affiliations:** 1 MRC Epidemiology Unit, University of Cambridge, Cambridge, UK; 2 Health Sciences School, University of Sheffield, Sheffield, UK

**Keywords:** Physical activity, Socioeconomic inequalities, Covid-19, Young people, Qualitative, United Kingdom

## Abstract

**Background:**

Adolescent physical activity levels are low and are shown to decline with age into adulthood. Emerging literature suggests these trends were exacerbated during the Covid-19 pandemic. We aimed to understand, from the perspective of adolescents living in deprived communities, whether the Covid-19 pandemic influenced their physical behaviour and explore their ideas for physical activity promotion moving forward.

**Methods:**

Purposive sampling was used to recruit older adolescents (13-18-year-old) living in one of the 20% most deprived areas in the UK, as defined by the UK Index of Multiple Deprivation. A mix of in-person and online one-to-one semi-structured interviews were conducted between July 2021- March 2022. Interviews were audio-recorded, transcribed verbatim and anonymised. Data were imported into Nvivo software and analysed drawing on Braun and Clarke’s six phases of thematic analysis.

**Results:**

The sample consisted of 16 adolescents and included a mix of genders. The following themes were generated during the data analysis: (1) Physical activity behaviour in everyday life (prepandemic), (2) The impact of Covid-19 on physical activity (during) and (3) Young people’s ideas about physical activity promotion (moving forward). Participants described themselves as inactive, with their activity limited to active travel, informal activity and physical education. Experiences of the pandemic were largely negative, impacting participants’ physical and mental health. Ideas around physical activity promotion ranged from the individual to the societal level.

**Conclusions:**

Our findings suggest the Covid-19 pandemic had a major impact on young people living in the context of socioeconomic deprivation. Physical activity promotion efforts should focus on school-based opportunities and the provision of safe and low-cost opportunities in socioeconomically deprived areas. As we aim to build back from the Covid-19 pandemic, supporting young people living in socioeconomically deprived communities should be prioritised.

**Supplementary Information:**

The online version contains supplementary material available at 10.1186/s12889-024-19777-z.

## Background

The health benefits of physical activity throughout the life course are well documented [1, 2]. Globally, the physical activity levels of 11-to-17-year-olds are low, with less than one in 10 adolescents meeting the physical activity guidelines of 60 min per day [3, 4]. These levels decline with age into adulthood, highlighting the importance of continued public heath efforts to increase and maintain physical activity during this life stage [5, 6].

Emerging literature suggests these trends were exacerbated during the Covid-19 pandemic. There is clear evidence for a decline in young people’s physical activity during the Covid-19 lockdowns [7]. The results of a recent systematic review and meta-analysis report a 17-minute reduction in young peoples’ daily moderate-to-vigorous physical activity levels during the Covid-19 pandemic [2]. Further evidence shows this decline was more prominent in older children and adolescents [3, 8]. Across global literature, this has been attributed to the closure of schools, sports clubs, and fitness centres and restrictions preventing the running of organised recreational activities [9, 10].

Globally, reports highlight inequalities experienced during the pandemic and its resulting restrictions [11]. This is attributed to the “syndemic” nature of pandemics, exacerbating existing inequalities in the social determinants of health [11, 12]. Regarding young people’s physical activity, socioeconomic background is reported to have impacted activity levels during Covid-19, with review level evidence highlighting those of a higher socioeconomic position to have been more active [7]. Socioeconomic and environmental factors likely contributed to disparities in health behaviours, including physical activity. For instance, areas of high deprivation typically have fewer recreational spaces and poorer neighbourhood safety so residents need to travel longer distances to activity areas [13–15]. People living in deprived areas were further disadvantaged by travel restrictions during the pandemic meaning that they could not travel to facilities outside their areas. Moreover, confined living arrangements for households on low incomes reduced opportunities for home-based physical activity [16].

Whilst the global literature provides insight on the impact of the pandemic, this varies depending on national strategies to curb the spread of the virus and a country’s societal circumstances entering the pandemic. Within the United Kingdom (UK), Impact Inquiry reports suggest young people were one of the groups worst affected by pandemic restrictions, which placed a huge strain on their health and well-being [17, 18]. Data from Sport England’s Active Lives Survey (2020–2021) aligns with global reports of a decrease in young people’s physical activity during the pandemic [19]. However, socioeconomic differences in physical activity remained similar to pre-pandemic values, suggesting inequalities remained constant.

Due to the nature of conducting research during a pandemic and under social distancing restrictions, conclusions have relied on self-reported survey data which have the potential to disguise socioeconomic inequalites [7, 20, 21]. Whilst this data show that young people from the UK across the socioeconomic spectrum became less active, previous research suggest those living in socioeconomically deprived communities likely had differing experiences and faced more barriers to being active [13]. However, this remains unclear in the UK context. As we recover from the pandemic, investment in equitable physical activity promotion is needed to mitigate the impact on health and health inequalities [22]. Developing our understanding of this from an adolescent perspective will help us learn from the pandemic and progress equitably toward national physical activity targets.

To achieves this, this study aims to understand, from the perspective of adolescents living in socioeconomically deprived communities, if and how the Covid-19 pandemic influenced their physical activity behaviour and explore their ideas for physical activity promotion moving forward.

## Methods

A qualitative approach was chosen to facilitate an in-depth exploration of the perspectives and experiences of young people living in the context of socioeconomic deprivation [20]. The Socio-Ecological Model was employed for the design and analysis of this study, acknowledging the complexity of physical activity behaviour and the multiple levels of influence [23]. Grounded in a subtle realist philosophy, the researchers aimed to assess the social world through the participants’ interpretations while recognising the influence of their subjective perceptions and applied research methods [24, 25].

This study was conducted following the Standards for Reporting in Qualitative Research (SRQR) (see Supplementary File 1) [26]. Ethical approval for the study was granted by the University of Cambridge School of Humanities and Social Sciences ethics committee (21.273).

### Participants and recruitment

Purposive sampling was used to recruit older adolescents (13–18) from socioeconomically deprived areas across UK. Area-level deprivation was used as a proxy for individual-level SEP to avoid stigmatising participants. In the first instance, schools across the East of England whose free school meal eligibility was in the highest quartile of the country, as defined by the Department of Education (> 18.8% of students), were contacted via email. Schools were provided with study information and asked if they would be willing to distribute this to their students. If no response was received, schools were sent a follow-up email and then contacted via phone.

Further strategies were developed due to the strain on schools in response to the Covid-19 pandemic and challenges in recruiting via this method. This included recruiting community groups (e.g. local youth groups or Scouts or Guides groups where young people participate in organised activities) across the East of England to facilitate participant recruitment. These groups were identified online and contacted via email if they were situated in one of the 20% most deprived areas in the UK, as defined by the Index of Multiple Deprivation (IMD) [27]. Community groups were also recruited via social media using a combination of direct messaging and study adverts. Recruited community groups were asked to share the study information with their group members.

Facebook and Instagram were used to run adverts targeted geographically towards adolescents living in deprived areas across the UK using the IMD (as described above). Finally, a snowball sampling technique was used where participants were offered a £15 Amazon voucher if they referred a friend and this friend took part.

Information was provided at the school, community group, and participant levels, outlining the purpose of the study and detailing what participation involved, including any potential risks and benefits, as well as the handling of the collected information. All participants recruited into the study provided informed consent, consent was also sought from the parent/guardian of participants < 16 years old. Participants provided the first 4 digits of their postcode to determine the IMD of their local area and the name of their school, this information was used as a proxy for Socioeconomic Position (SEP). As detailed above, only those living in the top 20% most deprived neighbourhood as determined by the IMD were eligible to participate. Using an information power approach, the final sample size was determined following an iterative process throughout data generation and analysis (see Supplementary File 2 for more information) [28] Table 1.

**Table 1:** Characteristics of study participants

Sample	Number of participants	Age	Gender	Deprivation position
Overall	16	Age range:13–18 Average age: 15.25	5 Male 9 Female 2 Prefer not to say	Deprivation level: top 20%
Cambridge and Fenland	6	18,16,13,13,14,16	3 Female 1 Male 2 Non-binary	Deprivation level: top 20%
Peterborough	5	18,16,15,13,13	5 Female	Deprivation level: top 10%
London	4	15, 15, 15, 16, 16	3 Male 1 Female	Deprivation level: top 20%
Birmingham	1	15	Male	Deprivation level: top 10%

### Data generation

One-to-one semi structured interviews were conducted between July 2021 and March 2022. The majority of interviews were conducted using Zoom video teleconferencing software, due to restrictions imposed during the pandemic. The researcher leading data collection later became immersed in a local community group (when restrictions had eased), attending weekly group sessions (in person) between December 2021 and April 2022. In-person interviews were conducted one-to-one with young people recruited through the community group. Interviews took place in a private space during their usually group session.

A single interview guide was developed which directed participants through a timeline of their physical activity behaviour before and during the UK lockdowns and ended with a discussion of ideas around physical activity promotion moving forward. Social distancing measures were still in place at the start of interview period, the semi-structured nature of the topic guide allowed the researcher to tailor each interview to the participant’s individual context and the timing of the interview in relation to the easing of restriction measures. Interview questions and prompts were guided by the Socio Ecological Model (the topic guide is available in Supplementary File 3).

All interviews were conducted by the same researcher who had experience conducting semi-structured interviews with young people from deprived backgrounds. Time was taken at the start of each interview to provide an overview of the session, check participants were happy to proceed and ease participants into the interview. With consent, interviews were digitally audio recorded. Each young person attended one interview lasting ~ 30-minutes to be considerate of participants’ time, avoid interview fatigue and encourage engagement [29].

After each interview, a written summary was developed and sent to participants to check and provide feedback [30]. Everyone received a £15 Amazon voucher to thank them for their time. Young people had the opportunity to opt into a Photovoice element of the study for an additional £10 Amazon voucher, which involved taking photos of their physical activity environment. Participants were made aware that data and postage costs associated with this would be covered by funding from the study. None of the participants opted into this option.

### Analysis

All interviews were transcribed verbatim, anonymised and imported into Nvivo software (Version 12 Pro, QSR International, Victoria, Australia).

Transcripts were analysed guided by Braun and Clarke’s (2006) six phases of thematic analysis: (1) familiarisation with the data, (2) generating initial codes, (3) searching for themes, (4) reviewing themes (5) defining and naming themes; and (6) writing the report [31]. The researcher actively coded the transcripts, developing themes through layered interpretations of the data [32].

This began with the author immersing themselves in the dataset, listening to the audio recordings and reading the interview transcripts. Data were coded using both inductive (data-driven) and deductive (theory-driven) approaches [31]. Initially, we prioritised an inductive approach to generate codes from the data, focusing on the narratives provided by young people. This approach foregrounded young people’s perspectives and experiences. Subsequently, we applied deductive coding to structure these narratives by applying a socioecological lens. This allowed us to examine the interrelationships between young people and their social, physical and policy environment [23]. Initial codes were sorted into themes, capturing multiple observations in the data. This involved re-focusing the analysis at the broader level, considering how different codes may combine into overarching themes and sub-themes.

Candidate themes and sub-themes were developed and reviewed by rereading all the collected extracts for each theme. Once satisfied these adequately captured the code data, they were further refined developing clear names and definitions for each theme. After a fully worked-out set of themes had been developed, the research team worked to produce a compelling story about the data which strongly reflected the views and narrative of young people and the aims of this study. This is presented in the following sections.

### Researcher characteristics and reflexivity

Adopting a subtle realist approach, the research team acknowledge the influence of their own subjective perception in their approach to the study. To maintain reflexivity the lead researcher and interviewer kept a journal documenting a self-critical account of the research process, including their immersion in a local community youth group. Throughout this time, they developed relationships with adolescents and staff members of the youth group, observing them in the context of their everyday lives. This undoubtedly influenced their approach to data analysis. Peer debriefing was used involving continual discussions about the research process and analysis, in addition to reflecting on researcher positionality and assumptions/subjectivities. More detail on researcher positionality and methods to enhance rigour and trustworthiness are outlined in Supplementary File 4.

### Public involvement and engagement

Methods for recruitment, interviewing and participant-facing materials were developed under the guidance of an existing youth advisory panel. Members of the group involved in this project were all between the ages of 13–18 years, to best represent the target age range of this project.

## Results

The following themes were generated during the data analysis: (1) Physical activity behaviour in everyday life (pre-pandemic), (2) The impact of Covid-19 on physical activity (during) and (3) Young people’s ideas about physical activity promotion (moving forward). A summary of each theme is outlined in Table 2 and Supplementary File 5 shows a thematic map for each theme.

**Table 2:** Description of themes with illustrative quotes

Themes	Sub-themes	Description of theme	Illustrative quotes
**Theme 1**: Physical activity in everyday life	**Subtheme 1**: What my physical activity looks like **Subtheme 2**: Factors influencing my physical activity **Subtheme 3**: Identity and gendered experiences of physical activity	During this theme young people described their physical activity behaviour before the Covid-19 pandemic, factors influencing their physical activity behaviour (focusing on barriers to physical activity) and their experience of physical activity in relation to gender and personal identity.	“I did two hours of PE at school but I didn’t do much apart from that, I just did like one a month of PT at Cadets, so once a month, and then I went on a couple of walks during the week but not too much or too long, so it wasn’t that much physical activity” (Male, 15y). “…but that’s like for after school and I know that a lot of people like wouldn’t be able to do stuff like that because obviously they have to get home like early or something like that and they have to get the bus and some people have job they have to do” (Male, 16y). “I don’t know, I just feel like for some girls you are just worried about what you look like and if people are judging you” (Female, 16).
**Theme 2**: The Impact of Covid-19 on Physical Activity	**Subtheme 1**: The impact on my physical activity behaviour **Subtheme 2**: The impact on my physical health **Subtheme 3**: The impact on mental health **Subtheme 4**: Physical activity and the return to school during Covid-19 pandemic	During this theme young people described their experiences of physical activity during the Covid-19 pandemic. This was discussed in relation to participants physical and mental health in addition to returning to school.	“I just stayed in bed all day (during lockdown). And when I went out and went to school (post lockdown), I had to walk for twenty minutes and my legs started to hurt” (Female, 18y). “I pulled my hamstring on my right leg, I think it was three times after lockdown. Because like I’d kick a football and I hadn’t been able to do that for like over 6 months” (Male, 16y). “I had like intrusive thoughts sometimes that I needed to like, to try and get rid of, so walking really helped” (Female, 18y).
**Theme 3**: Young people’s ideas about physical activity promotion	**Subtheme 1**: Ideas about physical activity promotion- individual **Subtheme 2**: Ideas about physical activity promotion- interpersonal **Subtheme 3**: Ideas about physical activity promotion- organisational **Subtheme 4**: Ideas about physical activity promotion- community **Subtheme 5**: Ideas about physical activity promotion- public policy	This theme outlines young people’s ideas for physical activity promotion moving forward. Discussions covered each level of the Socio Ecological Model	“I think like maybe promoting more sports clubs and stuff. I was thinking of joining a badminton club, some of them can be like kind of expensive but if they’re made a bit cheaper for students they might want to go more” (Female, 16y). “I think we need to help them (young people) with mental health and this would motivate them to do more physical activity. It isn’t really talked about you know, like the link between the two” (Female, 18y). “My sister in school has these things that are like challenges. And they gave, they give her rewards like and things, where she can do all the things with those points. And some of the challenges are physical activities, so maybe doing that is a good idea” (Female, 18y).

### Physical activity behaviour in everyday life (pre-pandemic)

#### What my physical activity looks like

The young people participating in this study often labelled themselves as inactive. They described being most active during the week, through school travel, housework/caring roles, unstructured activity (e.g. skateboarding at the park) and Physical Education (PE). All participants used active travel to commute to school, often combining waking with other transport modes such as the school bus. Active travel was further used for leisure activities, one participant described, *“It kind of depends*,* sometimes I walk*,* I just walk to the other villages*,* why not? I don’t really have another option” (Female*,* 16y)*.

PE was described as a key contributor to weekly activity, with participants experiencing limited opportunities to be active beyond this setting, due to the barriers highlighted below (subtheme 2). When outlining their physical activity in a “usual” week, one young person described,

*“Well*,* there is travel to and from school*,* probably like 5 minutes to walk to the bus stop and then the bus just takes me. But mainly 2 hours of PE in school and skating here (youth group) every Wednesday and Thursday” (Non-binary*,* 14y)*.

Females at the upper end of the age spectrum conveyed the importance of dance as part of the school curriculum, whereas males more often referenced football, *“Er*,* basically after school on weekdays just go out to play football with my mates…” (Male*,* 16y)*. Outside of school, males more frequently reported participating in unstructured activities, whereas females were more likely to take on an active role at home through household chores or caring for siblings, thereby reducing their sedentary time.

“*TV at my dad’s but at my mum’s my younger sister*,* having to watch her… I had to keep going out there to watch my little sister because my little sister loves going on the trampoline” (Female*,* 13y)*.

With the exception of a few participants living in close proximity to their school, most described being unable to participate in extra-curricular activities as they relied on the school bus to get home. Instead, participants engaged in informal physical activities such as playing football with their friends or going to the skatepark, *“We don’t do training […] Some people just bring their football and we just play on the field and run with it.” (Male*,* 16y)*.

#### Factors influencing my physical activity

A focus across participants was the barriers they faced to being active, including the previously mentioned transport barriers and having an after-school job. For most participants this meant they were unable to attend after school sports clubs. For example,

*“…but that’s like for after school and I know that a lot of people like wouldn’t be able to do stuff like that because obviously they have to get home like early or something like that and they have to get the bus and some people have job they have to do” (Male*,* 16y)*.

When discussing the lack of facilities in his local community, one participant described driving.

past a swimming pool on a school trip and how he wished facilities like this were available where he lived, *“like why isn’t that in my area because I love swimming*,* I love swimming a lot so I think they should make it like more local” (Male 15y)*. Building on this, the travel options available to many participants were described as being poor quality and dangerous which prevented them accessing physical activity opportunities. One participant explained, *“Like you can go on a bike but it’s like really dangerous because of how fast cars go down those roads” (Male*,* 14y).* Journey length using public transport was a further barrier, with participants reflecting on their limited access to clubs and facilities in the local community.

*“I guess when*,* the only times I see like after school clubs are like places that are quite far actually … ” (Male 15y)*.

The physical activity facilities that most participants could access were described as either of poor quality or in disrepair, with vandalism and safety concerns being major problems. Similar barriers were experienced in the school environment, with lack of and poor-quality equipment being a major focus in addition to limited activity choice, for example *“There’s like football and that’s all I know of that’s doing physical activity” (Female*,* 18y)*.

#### Identity and gendered experiences of physical activity

Being a ‘sporty’ person was identified as important for physical activity participation. Participants described being ‘sporty’ as someone who is athletic, enjoys sport and is good at it. Physical activity was associated with defining oneself or being seen as ‘sporty’ and being part of a ‘sporty’ friendship group. For example,

“*like there’s the group of people that do exercise like the football group and everybody else*,* you’re not really*,* don’t really do exercise or do anything like that. So*,* I think it’s so much that stereotype type*,* type of people of like*,* “Yeah*,* no*,* it’s not for me” (Female*,* 16y)*.

Among female participants, feeling self-conscious about participating in physical activity was frequently mentioned. One participant described, *“I wouldn’t personally want to like run around the streets or anything because I might see someone I know…” (Female*,* 16y)*. Concerns about skill level were reported to compound this, for example, *“Especially*,* yeah if you’re not good at the sport*,* like I think it’s another layer of like…” (Female*,* 18y).* Male narratives primarily focused on skill level but framed this positively as they described how they enjoyed being skilful and making the most of opportunities to display this skill to others.

*“You’ll be able to show off that you’re a good boxer or whatever*,* anything that you want to like*,* anything that you want to be a perk and yeah just make something of it you know” (Male*,* 16y).*

A further point of discussion was the “gendered” nature of sport and how this limited the activity opportunities available to young people, especially at school. For example,

*“Sports are all quite gendered*,* like boys play football*,* girls play netball in school. That’s one of the things that maybe just like I know quite a few boys who would love to play netball*,* but they can’t because they’re not allowed to” (Female*,* 13y)*.

This was a prominent narrative among participants identifying as non-binary who further discussed the restrictive nature of the school PE kit, *“The school PE kit we have it’s like you have to wear a skort and like a t-shirt*,* but the skorts are like really short compared to like boy’s shorts” (Non-binary*,* 13y).*

### The impact of covid-19 pandemic on physical activity

#### The impact on my physical activity behaviour

Participants discussed the impact of the Covid-19 pandemic and resultant lockdowns on their physical activity. Lack of routine was frequently referred to, with activities such as traveling to school being replaced with sedentary activities, including watching TV or playing video games. Upon returning to school, all participants noticed a stark change in their activity levels due to the increase in structure.

*“It was easier to be active because school was more structured instead of what it had been before (in the summer lockdown) so it…like I knew what my lessons were*,* then I can do my work*,* there’s like walking and stuff and had more of like a routine” (Female*,* 16y).*

Importantly, young people did not feel this applied to online learning. During the winter lockdowns, online learning became more structured and the work volume increased. Participants felt this had a negative impact on their physical activity due to the increase in sedentary screen time and the long duration of online classes allowing little time for movement during the day. One participant explained,

*“They (school) had even more work this time and we had lessons that lasted 2 hours and I barely had time to eat lunch. So I mean the first few weeks were the worst. I mean I once stayed ‘til 5 doing maths work because they put too much work” (Female*,* 13y).*

Some found it difficult to engage in schoolwork at home and had to return to school for supervised lessons. This had a positive impact on their physical activity behaviour due to the reintroduction of active travel to school. One participant described,

*“Some had*,* some people had to go to school because they weren’t doing the work because their parents wouldn’t make them. My mum made me do that because I was not doing work at home*,* she would come home from work and I was just sat watching TV. But then at least*,* at least I got to go out and do something and like I walked part of the way to school” (Male*,* 15y)*.

The omission of PE and dance from the online curriculum was thought to contribute to a decrease in physical activity. For example, one participant described how she stopped dancing during the lockdowns as her school did not set work for her dance class, the participant used the term “non-curriculum based work” to refer to non-exam based school work as she was preparing to take her General Certificate of Secondary Education exams (GCSEs).

*“Like they usually they set like non-curriculum based work for like all my other subjects*,* apart from dance*,* so like I didn’t do any dancing” (Female*,* 15y)*.

#### The impact on my physical health

Two participants shared their struggle of weight gain during the lockdowns. This was a real challenge to their personal identity and had a negative impact on their physical and mental health and relationships.

*“So I was like a floater*,* like a ghost or stuff*,* like I just followed like a group around the like the place so it was*,* I don’t know*,* I was just scared to speak to people because I feel felt like people were going to like make fun of me because the like me gaining weight was like noticeable as well. So I just didn’t want*,* I just didn’t want to speak because I was scared like people were going to start making fun of me and stuff” (Male*,* 16y)*.

A common theme among young people was finding the return to “normal” difficult (during periods of eased lockdown restrictions). They reported negative impacts on their physical health including fitness loss, injury and weight gain. This included breathing difficulties when returning to PE and feeling more out of breath than expected. Sports injuries were also reported.

*“I pulled my hamstring on my right leg*,* I think it was three times after lockdown. Because like I’d kick a football and I hadn’t been able to do that for like over 6 months” (Male*,* 16y)*.

#### The impact on my mental health

Some participants described how they had struggled with poor mental health and low mood, communicating a nuanced understanding of the interrelationship between physical and mental health in relation to physical activity. For males, this was primarily experienced as anger, which was exacerbated by being confined at home. One participant discussed how the anger issues he experienced before the Covid-19 pandemic became worse during the lockdowns.

*“Er…it probably affected my attitude because I was quite angry most of the time*,* I have anger problems so I just hit a window and my knuckles they had bent out of place” (Male*,* 16y).*

Feeling uncertain about the future and a lack of control was also described as negatively impacting mental health. For some young people, the meaning of and motivation for physical activity changed during the pandemic. Physical activity was often used as a coping mechanism, especially walking. One participant described, *“I had like intrusive thoughts sometimes that I needed to like*,* to try and get rid of*,* so walking really helped” (Female*,* 18y).*

#### Subtheme 4: physical activity and the return to school during Covid-19 pandemic

When returning to school, the introduction of one-way systems, separating the school by year group zones and the removal of classroom rotations was reported to impact incidental physical activity like walking between lessons and recreational activity at break times.

*“So we were constricted to our own zones*,* which meant that I wasn’t like playing ball and things like that as well. And because I’m top set in everything I was stuck in one classroom every day because they designated like a top set classroom*,* second set classroom and things like that*,* so I was just stuck in one classroom all day*,* just sitting there just waiting for my teacher to come” (Female*,* 13y).*

Participants further described how social distancing measures impacted PE/dance lessons. This included being restricted to certain zones and types of activities, wearing masks and not having access to sports equipment or changing facilities. The quote below provides an example,

*“At school it was like we had to go to school like on the days we had dance*,* we had to go in our like dance kit and then we’d do it like for an hour*,* and then we’d like carry on wearing it around the school*,* but like even then we had like boxes on the floor so we couldn’t do any like group work or like partners*,* and part of like the qualification is based on relationships in dance. And it was like we just couldn’t do that*,* and we had to wear masks the whole time*,* and the amount of people that had to sit down because they thought they were going to pass out*,* it was so bad” (Female*,* 15y).*

### Theme 3: young people’s ideas about how to promote physical activity

This final theme outlines participants’ recommendations for physical activity promotion moving forward. Interview questions specifically focused on each level of the Socio Ecological Model, reflected by the subthemes below.

#### Subtheme 1: ideas about physical activity promotion- individual

Narratives focusing on the individual centred around young people’s knowledge of the benefits of physical activity and identity in relation to being active. This was linked to earlier discussions around not identifying as “sporty” and how this impacted young people’s interest in being active.

*“I just think*,* erm well*,* like if you are not sporty I just don’t think you will be interested in doing any activity*,* so I am not really sure what you can do” (Non-binary*,* 14y).*

Many young people discussed promotion efforts to increase awareness of the physical and mental health benefits of physical activity, in addition to support for those with poor mental health. A further demonstration young people’s nuanced understanding of the relationship between physical and mental health.

*“I think we need to help them (young people) with mental health and this would motivate them to do more physical activity. It isn’t really talked about you know*,* like the link between the two” (Female*,* 18y).*

Recommendations included increasing awareness about the link between physical activity and mental health, for example, through school or social media channels targeting young people. Participants suggested providing online resources highlighting physical activity opportunities aimed at young people, such as adolescent only gym times and helplines to chat about how physical activity could impact their mental health.

#### Subtheme 2: ideas about physical activity promotion- interpersonal

When discussing promotion strategies, specific focus across participants was placed on peer relationships and the importance of friends as co-participants. Friends were described as facilitators in taking up opportunities and co-participation was suggested as a way to encourage more young people to be engaged in physical activity. Parents were recognised as important sources of support, but young people recognised that their parents had work or other competing life demands.

*“I think it’d be a lot easier if like our friends did it because there’s a lot more of us*,* whereas I don’t think*,* like parents have like jobs and everything so they would be like time-restricted as well*,* but if we have like friends and stuff then we could all do it a lot easier together and I feel like that would better” (Female*,* 16y).*

Participants outlined the importance of encouragement and guidance in helping young people gain autonomy over their physical activity. Emphasis was frequently placed on encouragement, but not enforcement, as enforcing physical activity could be counterproductive. This encouragement did not have to come from a particular source and any encouragement was seen as beneficial e.g. from teachers, friends, parents etc. One participant described,

*“I think they just need that little extra push through like schools or like their communities*,* or like however*,* whatever form you’d want it to take*,* yeah*,* just to like introduce them to difference activities they could do and then they can decide what they do and don’t want to do” (Female*,* 16y)*.

#### Subtheme 3: ideas about physical activity promotion- organisational

School-based promotion was a major focus and included expanding the PE curriculum and offering a greater range of activities. Linking back to their ideas around choice, young people often suggested increasing students’ autonomy during PE and proposed giving a selection of activities to choose from.

*“I think it’s like the*,* the opportunity to have a choice in PE*,* you know*,* if they*,* you know*,* if they have like a whole array of sports to choose from to do and people could like just have the choice I think that’d be great*,* because you know*,* you’ve been told*,* yeah next week we’re doing hockey*,* remember your shin guards and your mouth guard and*,* you’d just be like*,* I don’t want to do this*,* when you’d rather prefer to like be doing like a dance lesson or rounders even*,* something like that” (Female*,* 18y).*

Beyond this, participants agreed school-based promotion should focus on the school day in order to reach all students. Suggestions focused on break time provision, including providing sports equipment and opening up sports facilities for student use. Some suggested class-based activities with a competitive element or rewards for meeting physical activity targets.

*“My sister in school has these things that are like challenges. And they gave*,* they give her rewards like and things*,* where she can do all the things with those points. And some of the challenges are physical activities*,* so maybe doing that is a good idea” (Female*,* 18y).*

Contrasting earlier narratives about compulsory physical activity, participants suggested adding extra lessons to the PE curriculum. Some young people highlighted how, for older students, it was only possible to take part in PE if they chose it as an Advanced (A-) level subject. They thought that all A-level students should have the opportunity to participate in physical activity/PE, *“There’s no PE at school*,* so I guess making it optional instead of just not having the PE” (Female*,* 18y).*

Overall, discussions focused on having fun and how this was more likely to encourage young people to be active going forward. One participant described how when sport is fun, it doesn’t feel like exercise,

*“I feel like*,* I don’t know*,* in school*,* you know in PE lessons*,* if they found like more fun sports. Because then you don’t even feel like you’re doing exercise*,* like in our school something they did was athletics and no-one really enjoyed it because it’s just running” (Female*,* 16y).*

#### Subtheme 4: ideas about physical activity promotion- community

Discussions around community-based promotion focused on increasing the provision of opportunities and places to be active, for example:

*“Um*,* I think maybe just having more opportunities and places that you can be active because I know in my*,* where I live particularly there is nowhere for young people to do activities” (Non-binary*,* 13y).*

Relating back to transport barriers described in theme 1, many recommendations were made to develop better travel infrastructure with a focus on cycling, *“Well*,* they could start putting paths in to like neighbouring towns*,* because I know quite a few people that would love to go visit neighbouring towns” (Female*,* 16y).*

#### Subtheme 5: ideas about physical activity promotion- public policy

In general, participants were uncertain about the role of the government in physical activity promotion and discussed how young people might respond differently to promotion efforts.

*“I think a lot of people would respond differently to like how government*,* how the government would roll out different*,* you know*,* different campaigns there could be*,* yeah. So yeah I probably couldn’t give a*,* a generalised opinion on how I think…” (Female*,* 18y).*

Some suggested the government provide advice on physical activity, equipping young people with the knowledge and agency to be active. Relating to discussions at the community level, many proposed improving or developing physical activity facilities and adding equipment to parks. The cost of accessing activity clubs in the local community further discussed, with participants suggesting the government subsidise and/or increase the provision of free sports clubs.

*“I was thinking of joining a badminton club*,* some of them can be like kind of expensive but if they’re made a bit cheaper for students they might want to go more” (Female*,* 16y).*

## Discussion

This study aimed to understand, from the perspective of adolescents living in socioeconomically deprived communities, if and how the Covid-19 pandemic influenced their physical activity behaviour and their ideas about physical activity promotion moving forward. These perspectives are discussed below in the context of the broader literature base.

### Physical activity in everyday life (pre-pandemic)

Young people described having limited access to structured physical activity opportunities, adding contextual understanding to inequities observed in self-reported physical activity, a method which favours the recall of organised sports participation [21]. Participants explained environmental factors in the community such as poor active travel infrastructure, access to sports clubs (locally and after-school clubs) and cost as barriers to such opportunities (pre-pandemic). This is consistent with existing qualitative and quantitative review-level evidence that highlights socioeconomic disparities in the built environment [13, 14, 33]. The community factors mentioned above provide direction for the equitable implementation of physical activity opportunities moving forward.

Participants discussed how the gendered nature of sport and school clothing can act as a barrier to participation in physical activity. This aligns with existent evidence focusing on children, which highlights that school uniforms can be a barrier to physical activity participation among girls, children from ethnic and religious minorities, gender-diverse students, and those from socioeconomically disadvantaged backgrounds [34–36]. Feeling self-conscious emerged as a common theme among females, as evidenced by qualitative research foregrounding pressure to perform, anxiety related to body image, and the reinforcement of gender stereotypes, especially among females from socioeconomically deprived contexts [13]. These findings underscore the intersection of gender and socioeconomic disparities in physical activity, with significant implications for inclusivity.

### The impact of covid-19 on physical activity behaviour (during)

Young people’s accounts highlight the significant impact of the Covid-19 pandemic on their physical activity. This adds to self-reported survey data by showing the impact of the pandemic may have worsened trends in inactivity and exacerbated inequities in the UK [19]. these findings are consisted with claims that older adolescents and those from lower socioeconomic backgrounds were among the wort affected groups [7]. The absence of routine emerged as a major barrier to physical activity, echoing similar experiences of the pandemic reported by young people in low-income settings in the United States (US) [37]. These findings support the structured day hypothesis, which suggests obesogenic behaviours, including physical inactivity, are better regulated when young people have structured days such as school weekdays [38]. The removal of this structure has been linked with socioeconomic inequalities in young people’s health behaviour [39].

The adverse impact on young people’s mental health supports reports that the pandemic disproportionately affected the mental health of young people from lower socioeconomic conditions [40]. This shows the impact of the removal of school routine in a UK context, which had been associated with increased socioeconomic inequalities in mental health and wellbeing [39]. Young people’s discussions about the effects of the pandemic on their physical health mirror reports among the general adult population, where reduced physical activity and prolonged sedentary behaviour have been associated with loss of muscular and cardiorespiratory fitness and weight gain [41]. This is particularly concerning when considering that these effects may persist and worsen into adulthood.

Young people’s descriptions of the return to school reflect reports that schools in socioeconomically deprived areas were the worst affected, due to disparities in resources [42]. Our study highlights the implications of this for physical activity were especially stark, as schools were a primary source of physical activity among this group. Challenges faced by these schools appear similar beyond the UK context, with social distancing measures, access to a gymnasium, avoiding close contact and the use of uncleaned equipment reported as major barriers to PE [43]. This suggests the pandemic highlighted existent inequalities in school resources and the provision of physical activity opportunities.

### Young people’s ideas about how to promote physical activity (moving forward)

Moving forward, Young people provided direction for physical activity promotion across the multiple levels of the Socio Ecological Model [23]. This included an increased focus on the benefits of physical activity, specifically the mental health benefits and the provision of mental health services. Friends were described as a primary source of social support and facilitators in taking up physical activity opportunities. This adds to the broader qualitative evidence, where adolescents living in the context of socioeconomic deprivation are reported to rely more heavily on social support from friends than family members [13, 44, 45].

There was a focus on school-based physical activity implemented during the school day, highlighting the importance of the school setting in health promotion among young people of a lower SEP [13, 39]. The opportunity to practice numerous physical activities (during PE, school sports, break time and field trips) and autonomy over these was emphasised by participants and aligns with previous research highlighting the desire for increased autonomy among this age group [46, 47], [48] Further emphasis was placed on promoting fun, rather than competition, a dominant narrative among qualitative research [46]. For example, placing less focus on “traditional” games sport such as football and netball and more focus on promoting physical activity as part of daily life. This contracts similar research with this population in the US, suggesting cultural differences might impact experiences of leveraging a competitive environment in physical activity promotion [37].

At the community level, young people’s recommendations support those of Rossi et al., (2021) who suggest moving forward policymakers and city planners increase access to safe and movement-friendly environments, especially in socioeconomically deprived areas [7]. This includes the provision of safe active travel infrastructure connecting places of importance to young people [8, 48, 49]. This was emphasised during discussions at the policy level, which focused on the structural environmental provision and access to low cost/free physical activity opportunities. In addition to the provision of support services for young people including physical activity and mental health helplines.

### Strengths and limitations

Focusing on young people’s perspectives, this study adds in-depth, contextual understanding to existent evidence on physical activity behaviour during Covid-19. Exploring the experiences of young people progresses our understanding of inequalities in physical activity and the development of equitable physical activity promotion. Further strengths include the semi-structured interview format and the development of personal connections with participants recruited through a youth group setting, both of which allowed for the generation of nuanced insights on the interview topics and in the sharing of participants’ experiences.

The majority of the sample being from one region means the findings may not fully reflect experiences beyond this context. However, this is consistent with a qualitative approach [50]. Members of our Public Involvement and Engagement panel were generally of a higher SEP than those that we were aiming to recruit to this study which may have contributed to recruitment challenges and lack of engagement in the Photovoice element of the study. Interviews were conducted over a one-year period after the final UK lockdown, making it challenging for those in later interviews to recall their experiences. Moreover, it is important to acknowledge the possibility of social desirability bias in answering interview questions, especially during zoom interviews where it can be challenging to build a rapport.

### Recommendations for research and practice

Young people’s focus on school-based interventions and the provision of facilities in their community highlight target areas for the development of equitable physical activity strategies and policy level change. These include investment in school-based interventions implemented during the school day and the provision of low-cost opportunities and active travel infrastructure in socioeconomically deprived areas. In order to make physical activity more inclusive, further efforts are required to move away from a gender focus in sport. This extends to school uniform policy, where modifying student uniforms and PE clothing may represent a simple intervention to enhance physical activity [36].

The importance of routine was emphasised, suggesting a need to prioritise the provision of structured physical activity opportunities [38]. This highlights the potential for future research exploring the development of the structured day hypothesis within the adolescent population, as current literature has focused predominantly on children. Its broader application to research investigating inequalities in adolescents’ experiences of routine in relation to health behaviour could have significant implications for policy and practice. For example, this could lead to target investment in the provision of free sports clubs during school holidays and in school-based interventions, such as offering after-school sports clubs during lunch breaks, especially at schools with a high proportion of students from socioeconomically disadvantaged backgrounds.

Moving forward, concerted efforts are required to reverse the decline in physical activity and address worsening mental health stemming from the pandemic. Young people made multiple recommendations, including the provision of online resources for discovering physical activity opportunities tailored to their age group, as well as mental health services which emphasise the benefits of physical activity. As highlighted by young people in the study, initiatives to promote physical activity have the potential to positively impact mental health, and vice versa. Targeted social media campaigns were proposed as one avenue for further exploration. Future research into how to effectively engage friends as a primary source of social support may enhance the success of these strategies.

## Conclusions

This qualitative study of young people’s narratives suggests the Covid-19 pandemic may have contributed to trends in inactivity during adolescence and exacerbated inequalities. Moving forward, efforts to tackle inequalities and increase physical activity should include promoting a variety of school-based physical activity opportunities during the school day which focus on fun and the structural environmental regeneration of socioeconomically deprived areas to provide young people with safe and low-cost physical activity opportunities. As we build back from the Covid-19 pandemic, efforts to support the physical and mental health of young people living in the context of socioeconomic deprivation should be prioritised.

## Electronic supplementary material

Below is the link to the electronic supplementary material.

Supplementary Material 1

Supplementary Material 2

Supplementary Material 3

Supplementary Material 4

Supplementary Material 5

## Data Availability

The datasets generated and/or analysed during the current study are not publicly available due to the sensitive nature of the research, but are available from the corresponding author on reasonable request.

## Declarations

### Study design

Primary qualitative data collection and analysis.

## Abbreviations

UK: United Kingdom
SRQR: Standards for Reporting in Qualitative Research
IMD: Index of Multiple Deprivation
SEP: Socioeconomic Position
PE: Physical Education
GCSE: General Certificate of Secondary Education
