# Supplementary material for: Adolescents’ physical activity during and beyond the Covid-19 pandemic: a qualitative study exploring the experiences of young people living in the context of socioeconomic deprivation
Source: BMC Public Health. 2024 Oct 22;24:2450. doi: 10.1186/s12889-024-19777-z (PMC11494794; doi:10.1186/s12889-024-19777-z)
Supplement: Supplementary file 2 — Supplementary Material 2 [file 12889_2024_19777_MOESM2_ESM.docx]

**Supplementary File 2:** Application of the information power approach

The information power approach proposed by Malterud et al., (2016) was used to determine the sample size required in the present study.^1^ The approach identifies five items along different dimensions which impact the information power of a sample: (1) study aim, (2) sample specificity, (3) use of established theory, (4) quality of dialogue, and (5) analysis strategy. The more information a sample holds based on these criteria and relevant to the study aims, the lower the number of participants needed.^1^ Our application of this is outlined in the table below.

Table 1 Application of the items and dimensions of the information power approach to determining the appropriate sample size for this project^1^

| **Item** | **Dimension** | **Application** | **Impact on sample size** |
| --- | --- | --- | --- |
| Study aim | Narrow to broad | Specific study aim, topic (physical activity), context (Covid-19 pandemic) and implications (physical activity promotion moving forward) | Smaller |
| Sample specificity | Dense to sparse | Specified target group due to focus on socioeconomically deprived adolescents i.e. dense | Smaller |
| Established theory | Applied or not | Socioecological model used to guide interviews and analysis | Smaller |
| Quality of Dialogue | Strong or weak | Open, clear and relevant communication from research participants. Interviewer experienced in conducting semi-structured interviews with this population. | Smaller |
| Analysis strategy | Case or cross-case | Thematic cross-case analysis | Larger |

1. Malterud K, Siersma VD, Guassora AD. Sample size in qualitative interview studies: guided by information power. *Qualitative health research.* 2016;26(13):1753-1760.
