# Supplementary material for: Adolescents’ physical activity during and beyond the Covid-19 pandemic: a qualitative study exploring the experiences of young people living in the context of socioeconomic deprivation
Source: BMC Public Health. 2024 Oct 22;24:2450. doi: 10.1186/s12889-024-19777-z (PMC11494794; doi:10.1186/s12889-024-19777-z)
Supplement: Supplementary file 3 — Supplementary Material 3 [file 12889_2024_19777_MOESM3_ESM.docx]

**Supplementary File 3**

**Interview Introduction:**

Thank you for coming along today. I am Olivia, we spoke via email! The aim of this session is for us to have an informal chat about how the Covid-19 pandemic and your physical activity.

**Consent:**

- Remember PIS?
- We can stop the interview at anytime or you can leave the call if you feel uncomfortable.

You might remember that we sent you an information sheet about the project. You have provided consent to participate in this interview, and previously you indicated that you were happy to participate in an interview. Participating in this interview is completely up to you. If you are happy to take part then please stay, and if you do not want to take part, feel free to leave the call. You are free to leave the interview whenever you would like.

*****Check caregiver consent for <16*****

**(visual consent provided by a caregiver on the call)**

**Confidentiality:**

I am hoping to use a voice recorder to record this interview, so that I can remember what we have said (show to participant). Next, we will get someone to type out our entire discussion so that I can read it through. This will be for my use only. In any written documents we won’t use your name; instead we will say that ‘participant x said this.’

___________________________________

I’d love to hear all of your thoughts on every topic we discuss! However, if you do not wish to answer a question, that is absolutely fine; feel free to ask if we can go onto the next question.

___________________________________

Do you have any questions? Check participant is happy with the session being recorded.

******Start Recording******

***(If participant agrees)***

**Icebreaker- Set “Ground Rules” before going onto the main part of the interview:**

Let’s start the interview by setting some ground rules together, I will start and then we can alternate…

e.g.

- You can say “pass” if you don’t want to answer.
- You can take time to think before you answer.
- Speak up if I don't understand something.
- There are no right or wrong answers; say what you feel…

(Can be done interactively using zoom whiteboard or equivalent depending on what video platform is used).

**Main Body of the Interview**

Just to recap, the questions are all around physical activity. I will ask about whether you think the pandemic had impacted your physical activity, please feel free to say what you really think, even if you don’t think it’s what I might want to hear or if you think you have a different opinion to other people.

**Warm-up questions (just to get you use to this kind of interaction) *assess if needed**

- Firstly, just a little information about yourself:
- Can you start off by telling me your age and year at school?
- Something new you have done in lockdown? (Prompt- give own example if they are struggling)/ A fun fact about yourself? (Prompt- give own example if they are struggling)
- Use the conversation to lead onto interview questions about physical activity.

**General Physical Activity Questions**

1. What does the term physical activity mean to you? **Possible follow-up/prompts:** what different kinds of PA can you think of (active travel, sport, domestic etc).
2. Is being physically active important to you? **Possible follow-up/prompts:** why do you think this?

**Before Covid-19**

1. Can you talk me though your activity in a normal week before Covid? **Possible follow-up/prompts:** were you active everyday? What kind of activities did you do? Where you happy with your physical activity before the pandemic? School/ out of school etc.

**During Covid-19**

1. If you think back, did anything chance in terms of your physical activity during **First lockdown (summer), Second lockdown (winter-November) and third lockdown (start of this year)** the lockdowns? **Possible follow-up/prompts:**

- What activity
- Where
- With whom
- When you are active (time of day)
- Time outside etc…

And how when things opened up again… after the first lockdown, and then the second (so now).

**After Covid-19**

1. What do you think your “normal” activity will look like after the pandemic? **Possible follow-up/prompts:**

**-** Return to normal?

- Increase?

- Decrease?

- Have they tried new things they might carry on doing? E.g walking / running / cycling etc

1. What might help you to become more active after the pandemic? Discuss across the individual, interpersonal, school, community, public policy levels.

**Possible follow-up/prompts**: What could you/ your school/ your parents/ the government do to help?

**Conclusion of Interview**

That’s all the questions I have for you today. Thank you so much for taking the time to talk to me. I will send you a voucher for today’s session via email (check this is okay, offer postal option). Is there anything else you’d like to tell us about the things we talked about today? Or anything further you would like to discuss?

I will now go away and create a visual document providing a summary of what we have discussed today. I will send this to you to check whether you agree and allow you to edit any parts you disagree with.

If you think of any questions after we finish today you can contact me using my university email address (make sure they have address).

Thank you very much for your time and attention. I appreciate you sharing your thoughts and experiences with me.
