# Supplementary material for: Adolescents’ physical activity during and beyond the Covid-19 pandemic: a qualitative study exploring the experiences of young people living in the context of socioeconomic deprivation
Source: BMC Public Health. 2024 Oct 22;24:2450. doi: 10.1186/s12889-024-19777-z (PMC11494794; doi:10.1186/s12889-024-19777-z)
Supplement: Supplementary file 4 — Supplementary Material 4 [file 12889_2024_19777_MOESM4_ESM.docx]

**Supplementary File 4:** Researcher characteristics and reflexivity

Throughout this project, the lead researcher acknowledges their dual role as both interviewer and analyst, drawing on prior experience working with and conducting research among young people living in socioeconomically deprived contexts. Embracing a subtle realist approach, the research team acknowledges the impact of their own subjective perceptions on various aspects of the project, including the selection of research questions and methodologies, interactions with participants, and interpretation of data. This recognition extends to their chosen analytical approach, which highlights the personal involvement of the researcher in the analysis rather than striving for objectivity. Below, the lead researcher offers contextual information to assist readers in interpreting the data analysis and to ensure transparency in reporting the findings.

The development of this project was shaped by the researcher's personal interest in socioeconomic disparities in adolescent physical activity. This interest traces back to formative life experiences, including growing up with parents from markedly different socioeconomic backgrounds and attending schools in diverse demographic areas.
Her experiences of socioeconomic disparities extended into her university years, where she engaged in research with young people living in disadvantaged regions of Scotland while also tutoring young people from more privileged backgrounds for their public school entrance exams. Being the final project of her PhD thesis, the researcher found it difficult not to be influenced by findings for other contributing research projects.^1,2^ The researcher also acknowledges that the findings of her previous projects likely influenced their dialogue with participants.

As part of the APAD-C project, the researcher immersed herself in a local youth group, forming connections with both the young members and staff while observing their daily lives. This immersion undoubtedly shaped her approach to data analysis, enabling her to gain deeper insights from conversations with young people that might otherwise have remained superficial. Throughout the data analysis process, the researcher's understanding of participants' everyday contexts facilitated the extraction of nuanced meanings from the data, however, she acknowledges that this meaning is based on her subjective perception.

To maintain reflexivity during data interpretation, the researcher kept a reflexive journal, providing a self-critical account of the research and reflecting on internal and external dialogues. This reflexive process involved maintaining an audit trail documenting decisions and methodological choices made throughout the study. Peer debriefing was employed to enhance credibility, involving ongoing discussions with the supervisory team regarding the study's development and receiving feedback on all written materials, including the study protocol, analysis plans, draft interview schedules, and project chapters.

**Table 1:** The application of Lincoln and Guba’s (1985) trustworthiness criteria to the CHILL qualitative sub-study analysis.^3^

| **Criteria** | **Techniques and their application** |
| --- | --- |
| Credibility (internal validity) | Prolonged engagement with the transcripts   - Interviews were transcribed throughout data collection to allow the researcher to read and reflect throughout data collection. - Raw transcripts were engaged with by the led researcher throughout the analysis.   Triangulation   - Triangulation of researchers throughout the analysis. - Triangulation of participant viewpoints, collecting data from young people across different regions in the United Kingdom.   Peer Debriefing   - Analysis conducted as a research team (as detailed in manuscript, see sections 2.3 and 2.4) - Feedback from the research team was provided on all written documents including but not limited to: the study protocol, interview schedule, analysis plans and resulting research paper for publication.   Referential Adequacy   - An iterative approach was taken to data analysis. - Raw data, codes and themes have been stored to show their development   Negative case analysis   - Data contradicting explanations emerging from the data were considered and discussed. |
| Transferability (external validity) | Trick description   - The research process has been described in detail using the Standards for Reporting Qualitative Research (SRQR) (see Supplementary File 1) |
| Dependability (reliability) | Dependability audit   - Raw data, codes and themes were stored to show their development. - An audit was kept of the developing “story” of the data. - The process of enquiry was continually re-examined, including but not limited to: how the data is collected, how the data was kept and the accuracy of the data in addressing the research questions and in adapting to Covid-19 pandemic restrictions. |
| Confirmability (objectivity) | Confirmability audit   - The data collection and analysis process has been transparently reported following the SRQR guidelines. - An audit trail was kept detailing each stage of the data analysis and of the research team’s discussions throughout this process. - The studies limitations have been acknowledged in the main manuscript (see Section 4.4. Strengths and Limitations). - Data was appropriately managed, including but not limited to: participant information, interview recordings and verbatim transcripts of interviews. |
| All four criteria | Reflexivity   - The researcher leading the data collection and analysis kept a reflexive journal documenting their positionality, notes of specific assumptions/subjectivities and an audit trial documenting decisions and choices made throughout the study. |

**Reference**

1. Alliott, O., Ryan, M., Fairbrother, H. and van Sluijs, E., 2022. Do adolescents' experiences of the barriers to and facilitators of physical activity differ by socioeconomic position? A systematic review of qualitative evidence. *Obesity Reviews*, *23*(3), p.e13374.

2. Alliott, O., Fairbrother, H., Corder, K., Wilkinson, P., & van Sluijs, E. (2023). Do socioeconomic inequities arise during school-based physical activity interventions? An exploratory case study of the GoActive trial. *BMJ open*, *13*(3), e065953**.**

3. Lincoln Y, Guba E. *Naturalistic inquiry* Vol 1. Newberry Park, London, New Delhi: Sage Publications 1985.
