# Supplementary material for: Adolescents’ physical activity during and beyond the Covid-19 pandemic: a qualitative study exploring the experiences of young people living in the context of socioeconomic deprivation
Source: BMC Public Health. 2024 Oct 22;24:2450. doi: 10.1186/s12889-024-19777-z (PMC11494794; doi:10.1186/s12889-024-19777-z)

**Supplementary File 5:** Thematic maps

(1) Physical Activity Behaviour in Everyday Life (pre-pandemic)


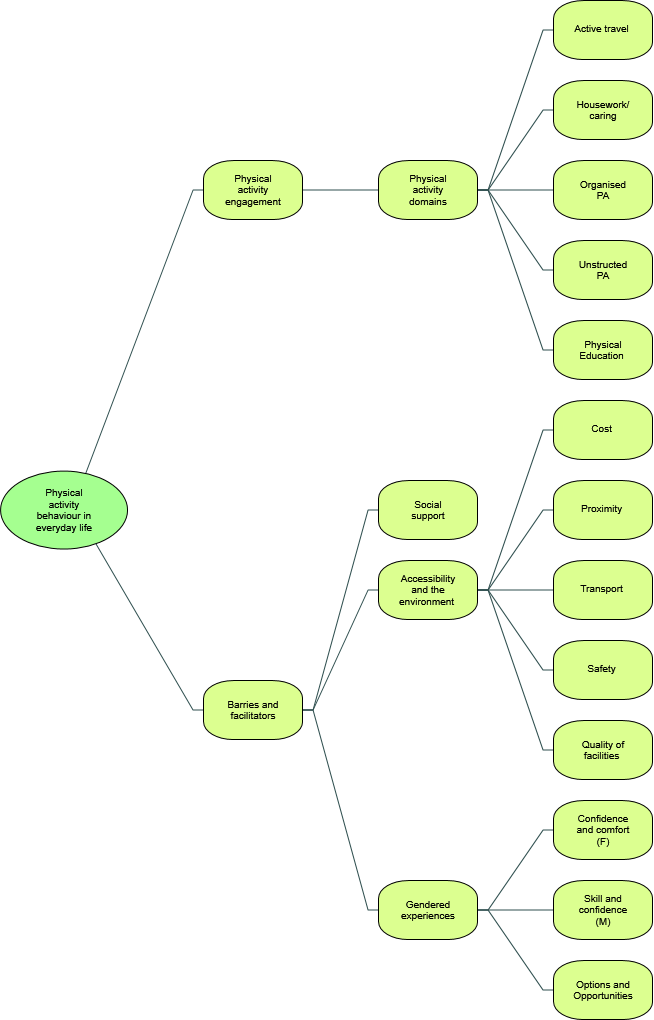


(2) The Impact of Covid-19 on Physical Activity (during)


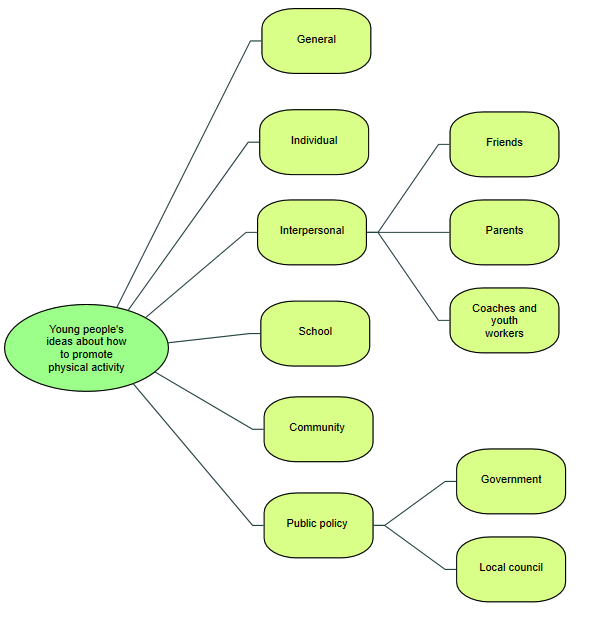


(3) Young People’s Ideas about Physical Activity Promotion (moving forward)


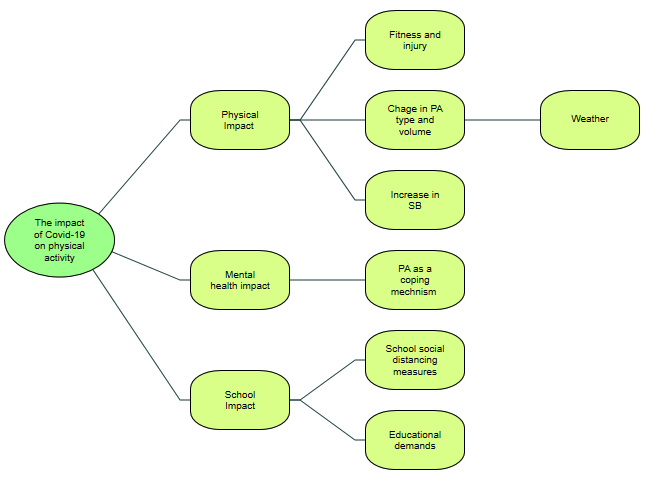

Supplement: Supplementary file 5 — Supplementary Material 5 [file 12889_2024_19777_MOESM5_ESM.docx]
